# Combined association of multiple chronic diseases and social isolation with the functional disability after stroke in elderly patients: a multicenter cross-sectional study in China

**DOI:** 10.1186/s12877-021-02439-9

**Published:** 2021-09-16

**Authors:** Xiangxiang Liu, Hong-jie Yu, Yan Gao, Jing Zhou, Mingchao Zhou, Li Wan, Feng Xiong, Jingpu Zhao, Qi-qiang He, Yulong Wang

**Affiliations:** 1 Shenzhen Second People’s Hospital, The First Affiliated Hospital of Shenzhen University, 518035 Shenzhen, China; 2 Shenzhen Dapeng New District Nan’ao People’s Hospital, 518121 Shenzhen, China; 3 School of Health Sciences, Wuhan University, 430071 Wuhan, China

**Keywords:** Elderly, Stroke, Social isolation, Functional disability, Multiple chronic diseases

## Abstract

**Background:**

Multiple chronic diseases (MCDs) and social isolation are independent risk factors related to stroke and disability, but it is unknown whether the combination of these two conditions resulted from aging-related to functional disability in stroke patients. This study aimed to probe the relationship between the combination of MCDs, social isolation, and functional disability after stroke in elderly patients.

**Methods:**

A multicenter and cross-sectional study was conducted in the Departments of Rehabilitation of 103 hospitals located in 23 cities across China. Stroke patients aged 60–90 years were selected for analysis. Demographic characteristics, lifestyles, and clinical information were investigated by questionnaires and medical records. MCDs (hypertension/ diabetes/ hyperlipidemia/heart disease/kidney disease) were categorized into three levels: 0, 1, and ≥ 2. Functional disability was assessed by the Barthel Index and categorized into four groups: no, mild, moderate, and severe disability. The multi-nominal logistic regression model was used to explore the independent and combined association of MCDs and social isolation with functional disability.

**Results:**

A total of 4046 elderly stroke patients (55 % males) were included in the final analysis. The prevalence of social isolation, MCDs ≥ 2, and severe disability increased with aging. In the fully adjusted model, patients with social isolation or MCDs had a higher risk of functional disability significantly than those without. Patients with social isolation combined MCDs ≥ 2 were 35 times (95 % CI: 18.89–64.69) more likely to suffer severe disability after stroke, and 8 times (95 % CI: 18.89–64.69) for moderate disability than those without social isolation and MCDs.

**Conclusions:**

MCDs, social isolation, and their combination were associated with a higher risk of functional disability after stroke in Chinese elderly patients. The elderly population should be encouraged to participate in more social activities, particularly in those with MCDs. Future secondary prevention and rehabilitation treatments to the functional ability of elderly stroke patients should underscore both social activity and the combined treatments of MCDs.

**Trial registration:**

NO: ChiCTR2000034067.

**Supplementary Information:**

The online version contains supplementary material available at 10.1186/s12877-021-02439-9.

## Background

Stroke, as the leading cause of death in China, affected over 12 million Chinese residents in 2013 [1]. It was estimated that over 2 million newly diagnosed stroke patients annually, and the incidence was projected to increase substantially [2]. Over 70 % of patients after stroke struggle to perform activities of daily living (ADL) and require substantial household and medical care [3]. A long-term cohort study (a mean of 13-year follow-up) described the disability trajectories and found a steep decrease in post-stroke functional ability [4]. Many studies have reported that the recurrence rate of stroke was approximately 10 % within one year [5], and with an increase to 12 % over five years [6]. The disability was more serious in recurrent stroke patients, two times higher than that of their first-ever counterparts in one year [7]. Some cost-effectiveness analyses have indicated that functional disability was one of the strong determinants of the medical costs of stroke patients independent of its subtype and severity [8, 9], while the recovery of functional ability can effectively decrease the medical costs and improve the quality of life of stroke patients.

Epidemiological evidence has shown that aging is the strongest non-modifiable risk factor for the morbidity of stroke and disability [10, 11]. The 2013 Global Burden of Disease study in China has shown that geriatric patients accounted for over 70 % of stroke deaths [12]. In general, the biological aging process is embedded with the interaction of multiple chronic diseases (MCDs) to form the pathogenesis of stroke [13]. A systematic review indicated that the prevalence of MCDs ranged from 55 to 98 % in elderly population [14]. In elderly stroke patients, the most prevalent chronic disease was hypertension (80 %); diabetes accounted for over 23 % [10]. Atrial fibrillation and chronic kidney disease were also associated with an increased risk of stroke [15, 16]. Furthermore, aging itself and its resulting MCDs have been shown to accelerate the simultaneous occurrence of stroke and functional disability [14]. Given that population aging continues to increase in China [17], studies addressing functional disability in elderly stroke patients are warranted.

In addition to MCDs, social activity is a potential pathway linking the aging process with stroke and functional disability. Commonly, social activity reflects the participation of activities that can enable connection and communication with other persons broadly, in the social milieu [18]. It has been found that over 40 % of the geriatric population suffers from social isolation (absence of social activity) due to the shifts in social relationships, including the geographic migration and death of family members [19]. Emerging evidence has shown that the presence of social isolation is associated with an increase in the risk of stroke [20] and disability [21]. Furthermore, it was found that either pre-stroke or post-stroke social activity was a robust predictor of stroke outcomes: pre-stroke social isolation was linked to a 40 % increase in adverse outcomes (including myocardial infarction, stroke recurrence, or death) after stroke [22], while Jansen’s study reported that 1-year post-stroke social isolation was still strongly associated with 3-year post-stroke disability [23].

Despite the independent association of MCDs and social isolation with stroke and disability has been suggested by previous studies [20, 21], little is known about whether the combination of these two conditions resulted from aging-related functional disability in elderly stroke patients. These two conditions reflected two aspects at the individual level: MCDs are a general indicator of physical health [14], while social activity reflects healthy social engagement and is strongly linked with mental health [20], but they interact with each other and cannot be treated separately. Based on a previous finding that social activity decreased the risk of MCDs in a European macro-regional study [24], we hypothesized that the combination of MCDs and social isolation can increase functional disability more than a single condition alone in elderly stroke patients. Thus, the objective of this study was to test the hypothesis in stroke patients aged 60–90 years using data from the Departments of Rehabilitation Medicine in 103 hospitals in China.

## Methods

### Study design and setting

This multicenter cross-sectional study was conducted between September 2018 and December 2019 in the Departments of Rehabilitation Medicine of 103 hospitals located in 23 cities around China. Originally, this study was designed to assess the reliability and validity of a pictorial scale (Longshi Scale) for disability assessment, which was constructed to reduce the time of disability evaluation in non-professional evaluation [25]. Subsequently, Longshi Scale was widely popularized at a national level to ensure its good application as the Barthel Index (BI), which is always regarded as the gold standard for assessing functional disability [26]. This study was approved by the Medical Ethics Committees of Shenzhen Second People’s Hospital (Project identification code: 20180926006). Written informed consent was obtained from all the inpatients or their proxies who agreed to participate in the study.

### Participants

This study recruited a total of 11,898 consecutive inpatients from the Departments of Rehabilitation Medicine. Among them, 1191 (10.0 %), 3426 (28.8 %), and 7281 (61.2 % patients were recruited from primary, secondary primary, and tertiary hospitals, respectively. For the purposes of this study, we only selected stroke patients aged 60–90 years for further analysis. The diagnosis of stroke was based on the 10th Reversion of the International Classification of Diseases (I60.x and I61.x for the hemorrhagic subtype; H34.1, I63.x, and I64.x for the ischemic subtype) [27]. For inclusion, patients were required to be conscious and able to read and answer questions. Those who suffered from deformities, mental illnesses, aphasias, and cognitive dysfunction were excluded. Additionally, patients who were participating simultaneously in other clinical studies were also excluded.

### Data collection

The sociodemographic characteristics, lifestyles, and clinical information of the participants were collected using questionnaires and medical records. Functional disability was assessed by trained healthcare practitioners (registered physicians, nurses, and therapists) at each collaborating hospital when patients receiving the rehabilitation treatment. All the data were recorded on electronic forms and uploaded to a specialized online system. Missing information was completed by requesting that the practitioner re-interview the participants.

### Measurement

Multiple chronic diseases: Our study considered five common chronic diseases: (1) hypertension, (2) diabetes, (3) hyperlipidemia, (4) heart disease, and (5) chronic kidney disease. Participants were asked “Were you diagnosed with any specific chronic disease by a physician in your lifetime?” and with only answer two options, “yes or no”. This question was widely used to assess MCDs [28]. Additionally, medical histories which reported any chronic disease, or treatment with related drugs or other therapies, were also recorded as “yes”. Eventually, self-report, medical history, and their combination were used to define chronic diseases. MCDs were classified based on the sum of categories: 0, 1, and ≥ 2.

Social activity: Participants were asked to report their social activities engagement during the last month before the stroke, involving four dimensions: (1) communication included deep conversations with relatives and friends by face-to-face or email; (2) leisure activities included chess and card entertainment, engaging in community clubs for singing and dancing together, playing outdoor exercise, fishing, hiking, and exercising with friends; (3) social affairs included voluntary or charitable work; and (4) education included some courses for health management and interest training. The investigation of social activity followed that used in a nationally representative longitudinal study [29]. Similarly, social activity was dichotomized by coding as “0” for no to indicate social isolation, “≥ 1” for yes to indicate social activity engagement.

Functional disability: The Barthel Index (BI) was used to assess functional disability. It consisted of 10 aspects of ADL and showed high reliability and validity in the Chinese population [26]: (1) feeding, (2) moving from wheelchair to bed and return, (3) personal toilet, (4) getting on and off the toilet, (5) self-bathing, (6) walking on a level surface, (7) ascending and descending on stairs, (8) dressing, (9) controlling bowel movements, and (10) controlling the bladder. Items 8 and 9 included four levels and were marked as 15, 10, 5, and 0 according to the independence of ADL, the items of 1, 4–7, and 10 included three levels and were marked as 10, 5, and 0, while items 2 and 3 only included two levels and were marked as 5 and 0, respectively. The total score ranged from 0 to 100, with a higher score indicating a higher functional ability. Based on previous studies, we divided participants into four groups: severe (BI ≤ 55), moderate (BI: 60–85), mild (BI: 90–95), and no disability (BI = 100) [26, 30].

### Statistical analysis

The demographic characteristics, current lifestyle, and clinical information were presented as numbers (%). Demographic characteristics included age (60–69, 70–79, and 80–90 *years*), gender (male and female), ethnicity (Han and Minority), marital status (married and single including divorced, widowed, and unmarried), annual household income (< 50,000, 50,000-100,000, and > 100,000 *yuan*), education level (primary and lower, middle school, high school, and college and higher), region (Eastern, Central, Western, and Northeast) [31], and hospital-level (primary, secondary, and tertiary). Current lifestyles which included drinking, smoking, and social activity were coded as “yes” and “no”, respectively. Clinical information included fall experience (none, fall without hurt, fall with light hurt, and fall with heavy hurt), stroke occurrence (first, second, and three or more), stroke type (ischemic, hemorrhagic, and combined), and MCDs.

Initially, an ordinal regression model was used to perform the association analysis; however, the parallel hypothesis test did not meet the requirement. Therefore, the independent associations of social activity and MCDs as well as their combination with functional disability, were examined using multi-nominal logistic regression models. Model 1 was the crude model; Model 2 adjusted the demographic information, lifestyles, hospital levels, and adjusted the clinical information. Given that aging influenced social isolation, functional disability, and MCDs [10, 11], the age-group (60–69, 70–79, and 80–90 years) stratified analyses regarding above-mentioned models were also performed. The multiple imputation method was used to handle the missing data with the imputation of five datasets. All analyses were conducted using SPSS software (Version 24.0. Armonk, NY: IBM Corp). A *P*-value of less than 0.05 was considered statistically significant. Given that falls with heavy hurt directly influenced mobility [32] and could suppress the impact of MCDs and social isolation on functional ability, a sensitivity analysis was conducted to exclude participants with heavy hurt fall experience and subsequently repeat the multi-nominal logistic regression model.

## Results

A total of 4046 elderly stroke patients aged 74.6 ± 8.5 years were included in this study, and their characteristics by age groups, are summarised in Table 1. Patients aged 60–69, 70–79, and 80–90 years accounted for about one-third, respectively. Among the total patients, 2231 (55.1 %) were male, 56 (1.4 %) were the minority, and over half were recruited from tertiary hospitals or eastern China. Patients who are currently drinking accounted for 7.2 %, and smoking accounted for 20.0 %. Approximately 75.0 % of patients suffered a stroke for the first time, and over 75.0 % of stroke were ischemic type. Over 40.0 % of patients had social isolation and suffered more than two chronic diseases. Moreover, 71.0 % of patients had a severe functional disability, 18.0 % had a moderate functional disability after stroke. Importantly, the prevalence of social isolation, MCDs ≥ 2, and severe functional disability increased with aging.

**Table 1:** Descriptive characteristics of 4046 elderly stroke patients according to age group

Characteristics, n (%)	60-69 y 1318 (%)	70-79 y 1365 (%)	80-90 y 1363 (%)
**Demographic**			
**Gender**			
Male	862 (65.4)	789 (57.8)	580 (42.6)
Female	456 (34.6)	576 (42.2)	783 (57.4)
**Ethnicity**			
Han	1290 (97.9)	1345 (98.5)	1355 (99.4)
Minority	28 (2.1)	20 (1.5)	8 (0.6)
**Marital status**			
Single (Widowed, Divorced, and Unmarried)	90 (6.8)	126 (9.2)	326 (23.9)
Married	1228 (93.2)	1239 (90.8)	1037 (76.1)
**Annual Household Income (*yuan*) **^**a**^			
<50,000	443 (33.6)	453 (33.2)	367 (26.9)
50,000-100,000	521 (39.5)	504 (36.9)	562 (41.2)
> 100,000	350 (26.6)	407 (29.8)	430 (31.5)
**Education Level **^**a**^			
Primary school and lower	371 (28.1)	422 (30.9)	520 (38.2)
Middle school	414 (31.4)	408 (29.9)	352 (25.8)
High school	328 (24.9)	316 (23.2)	265 (19.4)
College and higher	134 (10.2)	161 (11.8)	151 (11.1)
**Region**			
Eastern	769 (58.3)	838 (61.4)	953 (69.9)
Central	200 (15.2)	144 (10.5)	78 (5.7)
Western	272 (20.6)	336 (24.6)	315 (23.1)
Northeast	77 (5.8)	47 (3.4)	17 (1.2)
**Hospital Level** ^**a**^			
Primary	65 (4.9)	103 (7.5)	202 (14.8)
Secondary	366 (27.8)	421 (30.8)	622 (45.6)
Tertiary	861 (65.3)	816 (59.8)	498 (36.5)
**Drinking **^**a**^			
No	1174 (89.1)	1254 (91.9)	1308 (96.0)
Yes	142 (10.8)	104 (7.6)	51 (3.7)
**Smoking**^**a**^			
No	987 (74.9)	1046 (76.6)	1175 (86.2)
Yes	328 (24.9)	313 (22.9)	183 (13.4)
**Clinical**			
**Fall experience **^**a**^			
No	1107 (84.0)	1144 (83.8)	1173 (86.1)
Fall without hurt	42 (3.2)	45 (3.3)	29 (2.1)
Fall with light hurt	49 (3.7)	67 (4.9)	50 (3.7)
Fall with heavy hurt	37 (2.8)	50 (3.7)	83 (6.1)
**Stroke Occurrence **^**a**^			
First time	1012 (76.8)	1031 (75.5)	985 (72.3)
Two times	224 (17.0)	263 (19.3)	267 (19.6)
Three and more times	80 (6.1)	67 (4.9)	101 (7.4)
**Stroke type**			
Ischemic	890 (67.5)	1061 (77.7)	1186 (87)
Hemorrhagic	375 (28.5)	248 (18.2)	139 (10.2)
Combined	53 (4.0)	56 (4.1)	38 (2.8)
**Social isolation**			
Yes	646 (49.0)	586 (42.9)	402 (29.5)
No	672 (51.0)	779 (57.1)	961 (70.5)
**Multiple chronic diseases **^**b**^			
0	208 (15.8)	167 (12.2)	130 (9.5)
1	588 (44.6)	522 (38.2)	468 (34.3)
≥ 2	522 (39.6)	676 (49.5)	765 (56.1)
**Functional disability **^**c**^			
No	122 (9.3)	100 (7.3)	38 (2.8)
Mild	81 (6.1)	67 (4.9)	57 (4.2)
Moderate	307 (23.3)	234 (17.1)	170 (12.5)
Severe	808 (61.3)	964 (70.6)	1098 (80.6)

^a ^Has missing value. Annual household, 9 (0.2%); Education level, 204 (5.0%); Hospital level, 92 (2.3%); Drinking, 13 (0.3%); Smoking, 13 (0.3%); Fall experience, 170 (4.2%); Stroke occurrence, 16 (0.4%)

^b ^Chronic diseases include hypertension, diabetes, hyperlipidemia, heart disease, kidney disease

^c ^Measured by Barthel Index: severe disability (BI<55), moderate disability (BI: 60–85), mild disability (BI: 90–95), and no disability (BI=100)

We found that social isolation and MCDs were independently associated with functional disability after stroke (Table 2). After adjusting for demographic characteristics, current lifestyles, and clinical information, patients with social isolation were more likely to report severe (OR = 9.51, 95 % CI: 6.62–13.66, *P* < 0.001) and moderate (OR = 2.92, 95 % CI: 1.99–2.49, *P* < 0.001) disability relative to those without social isolation. Likewise, relative to patients with no MCDs, those with MCDs = 1 were more likely report severe (OR = 2.29, 95 % CI: 1.55–3.40, *P* < 0.001) and moderate disability (OR = 1.78, 95 % CI: 1.17–2.71, *P* = 0.008), so do the patients with MCD ≥ 2 (OR = 3.20, 95 % CI: 2.14–4.78, *P* < 0.001 for severe disability; OR = 2.61, 95 % CI: 1.70-4.00, *P* < 0.001 for moderate disability).

**Table 2:** The independent association of social isolation and multiple chronic diseases with the functional disability after stroke in elderly patients

	Model 1 ^**a**^			Model 2 ^**b**^		
	OR	95% CI	***P***	OR	95% CI	***P***
**Severe vs. no disability**						
**Social isolation**						
No	Ref			Ref		
Yes	9.56	(6.87-13.30)	<0.001	9.51	(6.62-13.66)	<0.001
**Multiple chronic diseases**						
0	Ref			Ref		
1	2.65	(1.84-3.81)	<0.001	2.29	(1.55-3.40)	<0.001
≥ 2	3.49	(2.41-5.06)	<0.001	3.20	(2.14-4.78)	<0.001
**Moderate vs. no disability**						
**Social isolation**						
No	Ref			Ref		
Yes	2.71	(1.90-3.87)	<0.001	2.92	(1.99-4.29)	<0.001
**Multiple chronic diseases**						
0	Ref			Ref		
1	1.91	(1.28-2.85)	0.002	1.78	(1.17-2.71)	0.008
≥ 2	2.57	(1.72-3.85)	<0.001	2.61	(1.70-4.00)	<0.001
**Mild vs. no disability**						
**Social isolation**						
No	Ref			Ref		
Yes	1.58	(1.00-2.48)	0.05	1.59	(0.98-2.58)	0.061
**Multiple chronic diseases**						
0	Ref			Ref		
1	1.65	(0.98-2.79)	0.06	1.54	(0.90-2.64)	0.117
≥ 2	1.75	(1.03-2.98)	0.039	1.66	(0.95-2.88)	0.073

^a ^Model 1 with *n* = 4046 was the crude model

^b ^Model 2 with *n* = 3573 adjusted for age group, gender, ethnicity, marital status, annual household income, education level, region, hospital level, drinking, smoking, fall experience, stroke occurrence, and stroke type

Table 3 displays the combined association of social isolation and MCDs with functional disability after stroke. The fully adjusted ORs of those with social isolation and MCDs ≥ 2 were 34.96 (95 % CI: 18.89–64.69, *P* < 0.001) for severe disability, 8.15 (95 % CI: 4.30-15.46, *P* < 0.001) for moderate disability, and 2.88 (95 % CI: 1.24–6.67, *P* = 0.014) for mild disability compared with those without social isolation or any chronic disease. Patients with social isolation and MCDs = 1 were also likely to report functional disability and the magnitude increased by its severity (OR = 2.45, 95 % CI: 1.07–5.61, *P* = 0.034 for mild; OR = 4.16, 95 % CI: 2.21–7.83, *P* < 0.001 for moderate; OR = 20.30, 95 % CI: 11.14–36.97, *P* < 0.001 for severe).

**Table 3:** The combined association of social isolation and multiple chronic diseases with the functional disability after stroke in elderly patients

Social isolation	Multiple chronic diseases	Model 1 ^**a**^			Model 2 ^**b**^		
		OR	95% CI	***P***	OR	95% CI	***P***
**Severe vs. no disability**							
**No**	0	Ref			Ref		
	1	2.98	(1.98-4.48)	<0.001	2.78	(1.75-4.43)	<0.001
	≥ 2	4.04	(2.67-6.11)	<0.001	3.68	(2.29-5.91)	<0.001
**Yes**	0	17.75	(8.16-38.64)	<0.001	16.54	(7.14-38.32)	<0.001
	1	20.32	(12.14-34.01)	<0.001	20.30	(11.14-36.97)	<0.001
	≥ 2	36.28	(20.74-63.46)	<0.001	34.96	(18.89-64.69)	<0.001
**Moderate vs. no disability**							
**No**	0	Ref			Ref		
	1	2.04	(1.31-3.17)	0.002	1.94	(1.19-3.14)	0.008
	≥ 2	2.46	(1.57-3.85)	<0.001	2.53	(1.54-4.14)	<0.001
**Yes**	0	3.98	(1.71-9.29)	0.001	3.91	(1.60-9.59)	0.003
	1	3.86	(2.20-6.77)	<0.001	4.16	(2.21-7.83)	<0.001
	≥ 2	7.75	(4.29-14.01)	<0.001	8.15	(4.30-15.46)	<0.001
**Mild vs. no disability**							
**No**	0	Ref			Ref		
	1	1.91	(1.08-3.39)	0.026	2.00	(1.06-3.77)	0.032
	≥ 2	2.09	(1.17-3.73)	0.013	2.14	(1.12-4.10)	0.021
**Yes**	0	3.30	(1.19-9.18)	0.022	3.88	(1.32-11.40)	0.014
	1	2.02	(0.96-4.24)	0.065	2.45	(1.07-5.61)	0.034
	≥ 2	3.16	(1.48-6.73)	0.003	2.88	(1.24-6.67)	0.014

^a^ Model 1 with *n* = 4046 was the crude model

^b^ Model 2 with *n* = 3573 adjusted for age group, gender, ethnicity, marital status, annual household income, education level, region, hospital level, drinking, smoking, fall experience, stroke occurrence, and stroke type

The age-stratified analysis regarding their independent associations (Table 4) indicated that social isolation was not significantly associated with moderate disability (adjusted OR = 1.72, 95 % CI: 0.78–3.79, *P* = 0.178), so did the association between MCDs and moderate disability. However, their association with severe disability was still significant, but the magnitude of OR for social isolation was the lowest in the 80–90 years group (OR = 5.48 in 80–90 years; 11.79 in 70–79 years; 11.61 in 60–69 years) and it for MCDs was the lowest in the 60–69 years group (OR = 1.60/1.98 for MCDs = 1/≥ 2 in 60–69 years; 3.28/5.07 in 70–79 years; 2.73/3.94 in 80–90 years).

**Table 4:** The independent association of social isolation and multiple chronic diseases with the functional disability after stroke in elderly patients by age groups

	60-69 y ^**a**^			70-79 y ^**a**^			80-90 y ^**a**^		
	OR	95% CI	***P***	OR	95% CI	***P***	OR	95% CI	***P***
**Severe vs. no disability**									
**Social isolation**									
No	Ref								
Yes	11.61	(6.22-21.69)	<0.001	11.79	(6.34-21.94)	<0.001	5.48	(2.62-11.5)	<0.001
**Multiple chronic diseases**									
0	Ref								
1	1.60	(0.84-3.03)	0.151	3.28	(1.76-6.12)	<0.001	2.73	(1.03-7.2)	0.043
≥ 2	1.98	(1.01-3.87)	0.048	5.07	(2.67-9.62)	<0.001	3.94	(1.56-9.94)	0.004
**Moderate vs. no disability**									
**Social isolation**									
No	Ref								
Yes	3.81	(2.00-7.28)	<0.001	3.34	(1.73-6.45)	<0.001	1.72	(0.78-3.79)	0.178
**Multiple chronic diseases**									
0	Ref								
1	1.37	(0.71-2.67)	0.352	2.21	(1.12-4.37)	0.022	2.18	(0.75-6.37)	0.152
≥ 2	1.94	(0.97-3.89)	0.061	3.48	(1.74-6.93)	<0.001	3.03	(1.09-8.41)	0.033
**Mild vs. no disability**									
**Social isolation**									
No	Ref								
Yes	2.31	(1.02-5.25)	0.045	1.23	(0.51-3.00)	0.643	1.01	(0.40-2.55)	0.987
**Multiple chronic diseases**									
0	Ref								
1	0.99	(0.44- 2.25)	0.986	2.48	(0.93-6.56)	0.068	1.65	(0.45-6.06)	0.451
≥ 2	0.71	(0.29-1.76)	0.457	2.83	(1.06-7.57)	0.039	2.61	(0.77-8.90)	0.125

^a^*n* = 1138 for 60-69 y, *n* = 1216 for 70-79 y, and *n* = 1219 for 80-90 y; adjusted for gender, ethnicity, marital status, annual household income, education level, region, hospital level, drinking, smoking, fall experience, stroke occurrence, and stroke type

The combined association of social isolation and MCDs with the functional disability after stroke was also stratified by age (Table 5). Likewise, their combination was significantly associated with severe and moderate disability in different age groups, and the magnitude of its OR in 70–79 years group was the highest and increased from moderate to severe disability simultaneously. However, the association of their combination with mild disability was not significant anymore in different age groups.

**Table 5:** The combined association of social isolation and multiple chronic diseases with the functional disability after stroke in elderly patients by age groups

Social isolation	Multiple chronic diseases	60-69 y ^**a**^			70-79 y ^**a**^			80-90 y ^**a**^		
		OR	95% CI	***P***	OR	95% CI	***P***	OR	95% CI	***P***
**Severe vs. no disability**										
**No**	0	Ref			Ref			Ref		
	1	2.20	(1.05-4.60)	0.036	2.92	(1.41-6.05)	0.004	4.55	(1.28-16.10)	0.019
	≥ 2	2.43	(1.12-5.27)	0.025	4.54	(2.15-9.58)	<0.001	6.18	(1.95-19.65)	0.002
**Yes**	0	29.14	(5.54-153.38)	<0.001	9.98	(3.12-31.96)	<0.001	23.11	(2.49-214.24)	0.006
	1	16.42	(6.21-43.43)	<0.001	33.96	(11.87-97.16)	<0.001	18.10	(5.14-63.79)	<0.001
	≥ 2	32.92	(11.32-95.77)	<0.001	58.05	(19.55-172.38)	<0.001	26.98	(7.92-91.87)	<0.001
**Moderate vs. no disability**										
**No**	0	Ref			Ref			Ref		
	1	1.46	(0.69-3.05)	0.321	2.45	(1.11-5.41)	0.027	3.51	(0.89-13.81)	0.072
	≥ 2	1.81	(0.84-3.91)	0.133	3.33	(1.48-7.48)	0.004	4.37	(1.24-15.41)	0.022
**Yes**	0	5.83	(1.05-32.41)	0.044	3.51	(0.97-12.68)	0.056	6.60	(0.61-71.11)	0.119
	1	3.81	(1.40-10.33)	0.009	6.39	(2.05-19.92)	0.001	4.18	(1.06-16.55)	0.042
	≥ 2	8.89	(3.02-26.18)	<0.001	13.57	(4.30-42.82)	<0.001	6.28	(1.66-23.77)	0.007
**Mild vs. no disability**										
**No**	0	Ref			Ref			Ref		
	1	1.21	(0.49-3.00)	0.687	3.20	(1.00-10.26)	0.051	3.73	(0.62-22.48)	0.151
	≥ 2	0.69	(0.25-1.93)	0.477	3.97	(1.23-12.82)	0.021	6.68	(1.28-34.78)	0.024
**Yes**	0	4.66	(0.65-33.45)	0.126	3.06	(0.50-18.70)	0.227	9.35	(0.65-135.41)	0.101
	1	1.61	(0.45-5.71)	0.462	4.00	(0.81-19.69)	0.088	3.19	(0.51-19.85)	0.214
	≥ 2	2.44	(0.60-9.86)	0.211	3.38	(0.63-18.17)	0.156	4.02	(0.69-23.38)	0.121

^a^*n* = 1138 for 60-69 y, *n* = 1216 for 70-79 y, and *n* = 1219 for 80-90 y; adjusted for gender, ethnicity, marital status, annual household income, education level, region, hospital level, drinking, smoking, fall experience, stroke occurrence, and stroke type

Results from the sensitivity analysis (Table S1 and S2) indicated that both independent and combined associations of social isolation and MCDs with the functional disability after stroke were robust after excluding patients who had a fall experience with heavy hurt.

## Discussion

Results from our study indicated that both social isolation and MCDs were independent risk factors for functional disability after stroke in elderly patients. Moreover, the hypothesis that a combination of these factors was associated with a greater extent of risk for functional disability than a single factor alone, was successfully assessed. The coexistence of social isolation and MCDs ≥ 2 was associated with a nearly 35 times higher risk for severe disability, 8 times higher risk for moderate disability than no social isolation combined with no MCDs.

This study, including 4046 elderly stroke patients from 103 clinical settings of 23 cities, was a nationally representative study in some degree. To the best of our knowledge, it is the first to address the combined association of social isolation and MCDs with functional disability in elderly population of China. We believe that these findings will provide insights that future prevention and intervention strategies for functional disability may integrate the social content with combined treatments of MCDs to achieve more effectiveness. However, the interpretation of these results also needs to consider the following limitations. First, the cross-sectional design of this study was restricted to draw the causal relationships among the variables. Second, the sampling method was non-random and included hospitals that collaborated with our departments. Nonetheless, the inherent bias may have been avoided since our study covered over 100 hospitals in 23 sites to ensure its generalisability. Additionally, our study only selected stroke patients aged 60–90 years, therefore our results may not be applicable to other aged populations. Third, the majority of variables were self-reported, recall bias thereby could not be avoided. Furthermore, considering that this study covered a large sample size and many organizations, the investigation of lifestyles selected only smoking and drinking as confounders, which might miss the information on other lifestyles relating to disability, including dietary behaviors, physical activity, and ex-smoking [33]. However, some information was investigated based on the standardized protocol to ensure study quality among different hospitals. For instance, self-reported MCDs were validated by their medical records, and functional disability was required to be evaluated by trained and experienced therapists. Fourth, our study only recorded the stroke type and occurrence and missed its severity, while the severity directly influences the functional ability [34, 35].

BI is the most popular method to measure functional disability in stroke patients [36]. Although the BI cut-off points for categorizing stroke patients with an unfavorable outcome were different from study to study, each study stressed the fact that the prevalence of functional disability was severe in stroke patients [36, 37]. There is a paucity of studies that consider elderly stroke patients in China. To the best of our knowledge, only Li et al. used the same classification criterion of BI (severe disability ≤ 55) in disabled elderly stroke patients with a small sample size (*n* = 158) and showed that the prevalence of severe disability was more than half [26]. The results of our study, similar to Li’s research, found that the prevalence of mild and severe disability was nearly 90 %. Moreover, our study found the prevalence of severe disability increased with aging (from 61.3 % in 60–69 years to 80.6 % in 80–90 years). Additionally, compared with studies in other countries using the same BI cut-off, the prevalence of severe disability in our study was more than double that of a Brazilian study of 260 middle-aged and elderly stroke survivors (31.5 %) [35], more than six times that of the Northern Manhattan Stroke Study of 340 elderly stroke patients [38].

The identification of risk factors for functional disability after stroke is key to the formulation of prevention and treatment strategies in elderly patients. In our study, MCDs status was found to be a risk factor for functional disability. Studies focusing on elderly stroke patients were limited, and we found some similar findings in a population with a single condition (geriatric or stroke) [39]. A recent systematic review of 3339 stroke patients from seven hospital-based cohort studies, indicated that MCDs were longitudinally associated with an 11 % increase in the risk of functional disability after stroke, irrespective of the different methods used for assessing MCDs and functional disability [39]. Meanwhile, a large cohort study including the Chinese geriatric population also showed that MCDs significantly increased the risk of functional disability by 117 % [40]. Another cohort study with a small sample size but with a mean age over 85 years found no significant association between them [41]. However, the findings of this study also identified a significant association between MCDs and functional disability in 80–90 years, but the magnitude of its OR was much lower than the other two young age groups (60–69 and 70–79 years). These findings suggested that the inconsistency of their association might be attributed to age. On the one hand, based on trajectory analysis [4], functional disability tended to be stable beyond the age of 80 years; on the other hand, the combination of MCDs and the age of over 80 years had a high probability of leading to death [40]. In fact, MCD status was inevitable to some extent in the aging process, [14] and nearly half of the participants in our study suffered from two and more chronic diseases, which was consistent with a recent systematic review [14]. Many possible explanations have addressed this issue. Sharing similar risk factors, including genetic, environmental, social, physiological, and psychosocial factors, was the common one [14, 42]. For example, chronic inflammation and oxidative stress played an important role in the initiation and progression of MCDs related to aging [42, 43]. Besides, the shared risk factors were more likely to be the mechanisms by which MCDs synergistically increased the functional disability [43]. Furthermore, another systematic review proposed that certain chronic disease clusters were internally and highly correlated [28]. As for stroke, we found that hypertension (77 %), diabetes (32 %), and heart diseases (33 %) were more common in stroke patients than the other two chronic diseases. A review similarly indicated that hypertension was the most important risk factor for stroke and sustained the highest population-attributable risk (34.6 %) among obesity, several classic chronic diseases (diabetes, hyperlipidemia, coronary artery disease, and arterial fibrillation), and lifestyles (smoking and physical inactivity) [44]. Thus, the prevention of post-stroke functional disability requires the control and management of hypertension, diabetes, and heart diseases.

Another strong risk factor for functional disability in our study was social isolation, which has been well documented in many cohort studies. [45–47] Although these studies were conducted in older adults without referring to stroke, they consistently indicated that social activity could reduce or delay the decline of functional disability caused by aging [45–47]. Social isolation may have a deleterious effect on neural networks and decreased musculoskeletal function, accelerating the functional disability related to aging [21]. In addition, many other behavioral and psychosocial pathways underlined the improvement in depression and cognitive decline associations with social activity; they all are important psychological risk factors for functional disability and stroke [22, 46]. Social networks change to smaller and close-knit ones during aging [19] and after stroke. [48] A clinical study has reported that baseline social isolation robustly predicted the stroke and prognosis outcome. [48] Thus, both primary prevention and secondary therapies for stroke and functional disability should consider the promotion of social engagement among older adults.

Importantly, the novel finding of our study is that the combination of social isolation and MCDs is associated with a much higher risk for functional disability than a single condition. Although the exact mechanisms regarding the relationship of their combination with functional disability have yet to be explained sufficiently, we can understand its plausibility from several dimensions: (1) both aging and stroke are the common risk factors for them [4, 7, 14, 20], although they represent social and biological levels, respectively; (2) social isolation may interact with MCDs through biological, behavioral, and neurological links. A previous study reported that a series of downstream biological changes, including abnormalities in cortisol secretion and a deterioration in immune and anti-oxidative stress functions, was easy to detect in people with social isolation [49–51]. From a behavioral point of review, social isolation constantly leads to the development of many unhealthy lifestyles, which accordingly increases the risk for MCDs [50]. As for the neurology aspect, a recent review synthesized the evidence and found that social isolation combined with other mental disorders (depression and anxiety) accelerated the activation of the hypothalamic-pituitary-adrenocortical axis and subsequently moderates the biological process [51]. Patients with low socioeconomic status are more likely to experience social isolation and loneliness [52], they also have higher risks of MCDs [53], which has been also demonstrated by a macro-regional analysis [24]. Furthermore, those with advanced socioeconomic status participating in more social activities and with fewer MCDs have more wiliness to absorb rehabilitation knowledge to decline the degree of functional disability [54]. Additionally, It should be noted that the OR of social isolation in the comparison between severe and no disability was still much higher than that of the other two comparisons (moderate vs. no disability; mild vs. no disability). Given that the intervention on social isolation through encouraging the participation of social activities was much more available than MCD management, future interventions aiming to increase functional ability and quality of life of elderly stroke patients should pay more attention to social activity.

## Conclusions

The findings of this study indicated that social isolation and MCDs were associated with the functional disability after stroke in Chinese elderly patients. In addition, the combination of these two risk factors is related to a much higher risk for functional disability than a single condition. Future secondary prevention and rehabilitation for functional disability in elderly stroke patients should underscore both social activity and the combined treatments of MCDs.

## Supplementary Information

**Additional file 1 **: **Table S1**. The independent association of social isolation and multiple chronic diseases with the functional disability after stroke in elderly patients without the heavy hurt fall experience. **Table S2**. The combined association of social isolation and multiple chronic diseases with the functional disability after stroke in elderly patients without the heavy hurt fall experience.

## Data Availability

The datasets used and/or analysed during the current study are available from the corresponding author on reasonable request.

## Abbreviations

MCDs: Multiple Chronic Diseases
ADL: Activities of Daily Living
OR: Odds Ratio
CI: Confidence Interval
