# Supplementary material for: Combined association of multiple chronic diseases and social isolation with the functional disability after stroke in elderly patients: a multicenter cross-sectional study in China
Source: BMC Geriatr. 2021 Sep 16;21:495. doi: 10.1186/s12877-021-02439-9 (PMC8447675; doi:10.1186/s12877-021-02439-9)
Supplement: Supplementary file 1 — Additional file 1 : Table S1. The independent association of social isolation and multiple chronic diseases with the functional disability after stroke in elderly patients without the heavy hurt fall experience. Table S2. The combined association of social isolation and multiple chronic diseases with the functional disability after stroke in elderly patients without the heavy hurt fall experience. [file 12877_2021_2439_MOESM1_ESM.docx]

**Table S1**. The independent association of social isolation and multiple chronic diseases with the functional disability after stroke in elderly patients without the heavy hurt fall experience

|  | **Total*** | | | **60-69 y*** | | |  | **70-79 y*** | | |  | **80-90 y*** | | |
| --- | --- | --- | --- | --- | --- | --- | --- | --- | --- | --- | --- | --- | --- | --- |
|  | **OR** | **95% CI** | ***P*** | **OR** | **95% CI** | ***P*** |  | **OR** | **95% CI** | ***P*** |  | **OR** | **95% CI** | ***P*** |
| **Severe vs. no disability** |  |  |  |  |  |  |  |  |  |  |  |  |  |  |
| **Social isolation** |  |  |  |  |  |  |  |  |  |  |  |  |  |  |
| No | Ref |  |  | Ref |  |  |  | Ref |  |  |  | Ref |  |  |
| Yes | 9.53 | (6.63-13.69) | <0.001 | 11.57 | (6.20-21.60) | <0.001 |  | 11.76 | (6.32-21.88) | <0.001 |  | 5.51 | (2.63-11.56) | <0.001 |
| **Multiple chronic diseases** |  |  |  |  |  |  |  |  |  |  |  |  |  |  |
| 0 | Ref |  |  | Ref |  |  |  |  |  |  |  |  |  |  |
| 1 | 2.31 | (1.56-3.42) | <0.001 | 1.59 | (0.84-3.01) | 0.159 |  | 3.33 | (1.78-6.22) | <0.001 |  | 2.78 | (1.05-7.35) | 0.039 |
| ≥ 2 | 3.24 | (2.17-4.84) | <0.001 | 2.00 | (1.02-3.93) | 0.044 |  | 5.19 | (2.73-9.85) | <0.001 |  | 3.99 | (1.58-10.08) | 0.003 |
| **Moderate vs. no disability** |  |  |  |  |  |  |  |  |  |  |  |  |  |  |
| **Social isolation** |  |  |  |  |  |  |  |  |  |  |  |  |  |  |
| No | Ref |  |  | Ref |  |  |  | Ref |  |  |  | Ref |  |  |
| Yes | 2.95 | (2.01-4.32) | <0.001 | 3.81 | (1.99-7.27) | <0.001 |  | 3.39 | (1.75-6.56) | <0.001 |  | 1.75 | (0.79-3.86) | 0.166 |
| **Multiple chronic diseases** |  |  |  |  |  |  |  |  |  |  |  |  |  |  |
| 0 | Ref |  |  | Ref |  |  |  |  |  |  |  |  |  |  |
| 1 | 1.79 | (1.18-2.73) | 0.007 | 1.41 | (0.72-2.76) | 0.311 |  | 2.17 | (1.10-4.28) | 0.026 |  | 2.2 | (0.75-6.41) | 0.149 |
| ≥ 2 | 2.59 | (1.69-3.98) | <0.001 | 1.99 | (0.99-4.00) | 0.053 |  | 3.33 | (1.67-6.64) | 0.001 |  | 2.96 | (1.06-8.21) | 0.038 |
| **Mild vs. no disability** |  |  |  |  |  |  |  |  |  |  |  |  |  |  |
| **Social isolation** |  |  |  |  |  |  |  |  |  |  |  |  |  |  |
| No | Ref |  |  | Ref |  |  |  | Ref |  |  |  | Ref |  |  |
| Yes | 1.52 | (0.93-2.48) | 0.093 | 2.31 | (1.01-5.27) | 0.048 |  | 1.24 | (0.51-3.01) | 0.640 |  | 0.90 | (0.35-2.31) | 0.828 |
| **Multiple chronic diseases** |  |  |  |  |  |  |  |  |  |  |  |  |  |  |
| 0 | Ref |  |  | Ref |  |  |  | Ref |  |  |  | Ref |  |  |
| 1 | 1.48 | (0.86-2.54) | 0.156 | 0.97 | (0.43-2.20) | 0.943 |  | 2.48 | (0.94-6.58) | 0.068 |  | 1.40 | (0.38-5.21) | 0.617 |
| ≥ 2 | 1.60 | (0.92-2.79) | 0.095 | 0.62 | (0.25-1.56) | 0.308 |  | 2.83 | (1.06-7.59) | 0.038 |  | 2.62 | (0.77-8.90) | 0.123 |

* n = 3424 for total, n = 1106 for 60-69 y, n = 1170 for 70-79 y, and n = 1148 for 80-90 y; adjusted for age group (only for total sample), gender, ethnicity, marital status, annual household income, education level, region, hospital level, drinking, smoking, fall experience, stroke occurrence, and stroke type.

**Table S2**. The combined association of social isolation and multiple chronic diseases with the functional disability after stroke in elderly patients without the heavy hurt fall experience

| **Social isolation** | **Multiple chronic diseases** | **Total*** | | |  | **60-69 y*** | | |  | **70-79 y*** | | |  | **80-90 y*** | | |
| --- | --- | --- | --- | --- | --- | --- | --- | --- | --- | --- | --- | --- | --- | --- | --- | --- |
|  |  | **OR** | **95% CI** | ***P*** |  | **OR** | **95% CI** | ***P*** |  | **OR** | **95% CI** | ***P*** |  | **OR** | **95% CI** | ***P*** |
| **Severe vs. no disability** | | | | | | | | | | | | | | | | |
| **No** | 0 | Ref |  |  |  | Ref |  |  |  | Ref |  |  |  | Ref |  |  |
|  | 1 | 2.78 | (1.75-4.43) | <0.001 |  | 2.17 | (1.04-4.56) | 0.040 |  | 2.92 | (1.40-6.07) | 0.004 |  | 4.61 | (1.30-16.38) | 0.018 |
|  | ≥ 2 | 3.75 | (2.33-6.03) | <0.001 |  | 2.47 | (1.14-5.37) | 0.022 |  | 4.66 | (2.20-9.86) | <0.001 |  | 6.32 | (1.98-20.13) | 0.002 |
| **Yes** | 0 | 16.6004 | (7.18-38.58) | <0.001 |  | 29.10 | (5.53-153.25) | <0.001 |  | 9.91 | (3.09-31.79) | <0.001 |  | 23.44 | (2.53-21.57) | 0.006 |
|  | 1 | 20.5003 | (11.26-37.45) | <0.001 |  | 16.32 | (6.17-43.22) | <0.001 |  | 34.48 | (12.03-98.84) | <0.001 |  | 18.58 | (5.26-65.68) | <0.001 |
|  | ≥ 2 | 35.33 | (19.07-65.45) | <0.001 |  | 33.04 | (11.34-96.31) | <0.001 |  | 58.50 | (19.67-173.95) | <0.001 |  | 27.51 | (8.06-93.94) | <0.001 |
| **Moderate vs. no disability** | | | | | | | | | | | | | | | | |
| **No** | 0 | Ref |  |  |  | Ref |  |  |  | Ref |  |  |  | Ref |  |  |
|  | 1 | 1.96 | (1.20-3.18) | 0.007 |  | 1.50 | (0.71-3.14) | 0.289 |  | 2.45 | (1.11-5.42) | 0.026 |  | 3.50 | (0.89-13.77) | 0.073 |
|  | ≥ 2 | 2.48 | (1.51-4.08) | <0.001 |  | 1.83 | (0.84-3.96) | 0.128 |  | 3.16 | (1.40-7.11) | 0.005 |  | 4.25 | (1.20-15.00) | 0.025 |
| **Yes** | 0 | 3.91 | (1.59-9.61) | 0.003 |  | 5.66 | (1.01-31.69) | 0.048 |  | 3.62 | (1.00-13.08) | 0.050 |  | 6.65 | (0.62-71.64) | 0.118 |
|  | 1 | 4.18 | (2.22-7.89) | <0.001 |  | 3.86 | (1.42-10.50) | 0.008 |  | 6.19 | (1.98-19.34) | 0.002 |  | 4.26 | (1.08-16.87) | 0.039 |
|  | ≥ 2 | 8.17 | (4.30-15.52) | <0.001 |  | 9.08 | (3.08-26.82) | <0.001 |  | 13.47 | (4.27-42.52) | <0.001 |  | 6.19 | (1.63-23.44) | 0.007 |
| **Mild vs. no disability** | | | | | | | | | | | | | | | | |
| **No** | 0 | Ref |  |  |  | Ref |  |  |  | Ref |  |  |  | Ref |  |  |
|  | 1 | 1.96 | (1.04-3.69) | 0.038 |  | 1.19 | (0.48-2.96) | 0.710 |  | 3.20 | (1.00-10.27) | 0.051 |  | 3.51 | (0.58-21.14) | 0.170 |
|  | ≥ 2 | 2.08 | (1.09-3.98) | 0.027 |  | 0.63 | (0.22-1.78) | 0.379 |  | 3.98 | (1.24-12.86) | 0.021 |  | 6.45 | (1.25-33.36) | 0.026 |
| **Yes** | 0 | 3.87 | (1.32-11.39) | 0.014 |  | 4.96 | (0.70-35.37) | 0.110 |  | 3.05 | (0.50-18.68) | 0.227 |  | 8.72 | (0.60-126.79) | 0.113 |
|  | 1 | 2.21 | (0.95-5.11) | 0.065 |  | 1.63 | (0.46-5.79) | 0.448 |  | 4.02 | (0.82-19.79) | 0.088 |  | 2.15 | (0.33-14.18) | 0.425 |
|  | ≥ 2 | 2.71 | (1.16-6.32) | 0.021 |  | 2.06 | (0.49-8.72) | 0.327 |  | 3.38 | (0.63-18.21) | 0.156 |  | 3.87 | (0.67-22.35) | 0.131 |

* n = 3424 for total, n = 1106 for 60-69 y, n = 1170 for 70-79 y, and n = 1148 for 80-90 y; adjusted for age group (only for total sample), gender, ethnicity, marital status, annual household income, education level, region, hospital level, drinking, smoking, fall experience, stroke occurrence, and stroke type.
